# Supplementary material for: The use of artificial intelligence tools in cancer detection compared to the traditional diagnostic imaging methods: An overview of the systematic reviews
Source: PLoS One. 2023 Oct 5;18(10):e0292063. doi: 10.1371/journal.pone.0292063 (PMC10553229; doi:10.1371/journal.pone.0292063)
Supplement: S1 Table — (DOCX) [file pone.0292063.s002.docx]

S1 Table - Database search strategy.

| **Database** | **Search (January the 12st, 2022)** | **References** |
| --- | --- | --- |
| **PubMed** | #1 - "diagnosable"[All Fields] OR "diagnosi"[All Fields] OR "diagnosis"[MeSH Terms] OR "diagnosis"[All Fields] OR "diagnose"[All Fields] OR "diagnosed"[All Fields] OR "diagnoses"[All Fields] OR "diagnosing"[All Fields] OR "diagnosis"[MeSH Subheading] OR ("diagnosable"[All Fields] OR "diagnosi"[All Fields] OR "diagnosis"[MeSH Terms] OR "diagnosis"[All Fields] OR "diagnose"[All Fields] OR "diagnosed"[All Fields] OR "diagnoses"[All Fields] OR "diagnosing"[All Fields] OR "diagnosis"[MeSH Subheading]) OR "Diagnoses and Examinations"[All Fields] OR "Examinations and Diagnoses"[All Fields] OR "Postmortem Diagnosis"[All Fields] OR "diagnoses postmortem"[All Fields] OR "diagnosis postmortem"[All Fields] OR "Postmortem Diagnoses"[All Fields] OR "Antemortem Diagnosis"[All Fields] OR "Antemortem Diagnoses"[All Fields] OR "diagnoses antemortem"[All Fields] OR "diagnosis antemortem"[All Fields] OR "Oral cancer diagnosis"[All Fields] OR "computer-aided diagnosis"[All Fields] OR "Cancer Early Detection"[All Fields] OR "Cancer Screening"[All Fields] OR "screening cancer"[All Fields] OR "non-invasive screening"[All Fields] OR "Cancer Screening Tests"[All Fields] OR "Cancer Screening Test"[All Fields] OR ("early detection of cancer"[MeSH Terms] OR ("early"[All Fields] AND "detection"[All Fields] AND "cancer"[All Fields]) OR "early detection of cancer"[All Fields] OR ("screening"[All Fields] AND "test"[All Fields] AND "cancer"[All Fields])) OR "screening tests cancer"[All Fields] OR "test cancer screening"[All Fields] OR "tests cancer screening"[All Fields] OR "Early Diagnosis of Cancer"[All Fields] OR "Cancer Early Diagnosis"[All Fields]  #2 - ("algorithm s"[All Fields] OR "algorithmic"[All Fields] OR "algorithmically"[All Fields] OR "algorithmics"[All Fields] OR "algorithmization"[All Fields] OR "algorithms"[MeSH Terms] OR "algorithms"[All Fields] OR "algorithm"[All Fields] OR ("algorithm s"[All Fields] OR "algorithmic"[All Fields] OR "algorithmically"[All Fields] OR "algorithmics"[All Fields] OR "algorithmization"[All Fields] OR "algorithms"[MeSH Terms] OR "algorithms"[All Fields] OR "algorithm"[All Fields]) OR "intelligence artificial"[All Fields] OR "Computational Intelligence"[All Fields] OR "intelligence computational"[All Fields] OR "Machine Intelligence"[All Fields] OR "intelligence machine"[All Fields] OR "Computer Reasoning"[All Fields] OR "reasoning computer"[All Fields] OR "ai artificial intelligence"[All Fields] OR "computer assisted diagnosis cad"[All Fields] OR "CAD"[All Fields] OR "diagnosis computer assisted"[All Fields] OR "computer-assisted diagnosis"[All Fields] OR "computer assisted diagnosis"[All Fields] OR "Computer-Assisted Diagnoses"[All Fields] OR "diagnoses computer assisted"[All Fields] OR "Computer Vision Systems"[All Fields] OR "Computer Vision System"[All Fields] OR "system computer vision"[All Fields] OR "systems computer vision"[All Fields] OR ("Artificial Intelligence"[MeSH Terms] OR ("artificial"[All Fields] AND "intelligence"[All Fields]) OR "Artificial Intelligence"[All Fields] OR ("vision"[All Fields] AND "system"[All Fields] AND "Computer"[All Fields])) OR ("Artificial Intelligence"[MeSH Terms] OR ("artificial"[All Fields] AND "intelligence"[All Fields]) OR "Artificial Intelligence"[All Fields] OR ("vision"[All Fields] AND "systems"[All Fields] AND "Computer"[All Fields])) OR ("Artificial Intelligence"[MeSH Terms] OR ("artificial"[All Fields] AND "intelligence"[All Fields]) OR "Artificial Intelligence"[All Fields] OR ("knowledge"[All Fields] AND "acquisition"[All Fields] AND "Computer"[All Fields])) OR ("Artificial Intelligence"[MeSH Terms] OR ("artificial"[All Fields] AND "intelligence"[All Fields]) OR "Artificial Intelligence"[All Fields] OR ("acquisition"[All Fields] AND "knowledge"[All Fields] AND "Computer"[All Fields])) OR "knowledge representation computer"[All Fields] OR ("Artificial Intelligence"[MeSH Terms] OR ("artificial"[All Fields] AND "intelligence"[All Fields]) OR "Artificial Intelligence"[All Fields] OR ("knowledge"[All Fields] AND "representations"[All Fields] AND "Computer"[All Fields])) OR ("Artificial Intelligence"[MeSH Terms] OR ("artificial"[All Fields] AND "intelligence"[All Fields]) OR "Artificial Intelligence"[All Fields] OR ("representation"[All Fields] AND "knowledge"[All Fields] AND "Computer"[All Fields])) OR "Computer Neural Network"[All Fields] OR "Computer Neural Networks"[All Fields] OR ("neural networks, computer"[MeSH Terms] OR ("neural"[All Fields] AND "networks"[All Fields] AND "Computer"[All Fields]) OR "Computer Neural Networks"[All Fields] OR ("network"[All Fields] AND "Computer"[All Fields] AND "neural"[All Fields])) OR ("neural networks, computer"[MeSH Terms] OR ("neural"[All Fields] AND "networks"[All Fields] AND "Computer"[All Fields]) OR "Computer Neural Networks"[All Fields] OR ("networks"[All Fields] AND "Computer"[All Fields] AND "neural"[All Fields])) OR "neural network computer"[All Fields] OR "models neural network"[All Fields] OR "model neural network"[All Fields] OR "network model neural"[All Fields] OR ("neural networks, computer"[MeSH Terms] OR ("neural"[All Fields] AND "networks"[All Fields] AND "Computer"[All Fields]) OR "Computer Neural Networks"[All Fields] OR ("network"[All Fields] AND "models"[All Fields] AND "neural"[All Fields])) OR "Neural Network Model"[All Fields] OR "Neural Network Models"[All Fields] OR "Computational Neural Networks"[All Fields] OR "Computational Neural Network"[All Fields] OR ("neural networks, computer"[MeSH Terms] OR ("neural"[All Fields] AND "networks"[All Fields] AND "Computer"[All Fields]) OR "Computer Neural Networks"[All Fields] OR ("network"[All Fields] AND "computational"[All Fields] AND "neural"[All Fields])) OR "networks computational neural"[All Fields] OR "neural network computational"[All Fields] OR "neural networks computational"[All Fields] OR "artificial neural network"[All Fields] OR "convolutional neural network"[All Fields] OR "Deep Learning"[All Fields] OR "learning deep"[All Fields] OR "Hierarchical Learning"[All Fields] OR "learning hierarchical"[All Fields] OR "Machine Learning"[All Fields] OR "learning machine"[All Fields] OR "Transfer Learning"[All Fields] OR "learning transfer"[All Fields]) AND ("asynchronous service models"[All Fields] OR "remote technologies"[All Fields] OR "remote technology"[All Fields] OR "virtual platform"[All Fields] OR "virtual platforms"[All Fields] OR "Mobile-phone-based screening"[All Fields] OR "Mobile Applications"[All Fields] OR "application mobile"[All Fields] OR "applications mobile"[All Fields] OR "Mobile Application"[All Fields] OR "Mobile Apps"[All Fields] OR "app mobile"[All Fields] OR "apps mobile"[All Fields] OR "Mobile App"[All Fields] OR "Portable Electronic Apps"[All Fields] OR ("Mobile Applications"[MeSH Terms] OR ("mobile"[All Fields] AND "applications"[All Fields]) OR "Mobile Applications"[All Fields] OR ("app"[All Fields] AND "portable"[All Fields] AND "electronic"[All Fields])) OR (("appl plant sci"[Journal] OR "apps"[All Fields]) AND ("portability"[All Fields] OR "portable"[All Fields] OR "portables"[All Fields]) AND ("electronical"[All Fields] OR "electronically"[All Fields] OR "electronics"[MeSH Terms] OR "electronics"[All Fields] OR "electronic"[All Fields])) OR ("Mobile Applications"[MeSH Terms] OR ("mobile"[All Fields] AND "applications"[All Fields]) OR "Mobile Applications"[All Fields] OR ("electronic"[All Fields] AND "app"[All Fields] AND "portable"[All Fields])) OR (("electronical"[All Fields] OR "electronically"[All Fields] OR "electronics"[MeSH Terms] OR "electronics"[All Fields] OR "electronic"[All Fields]) AND ("appl plant sci"[Journal] OR "apps"[All Fields]) AND ("portability"[All Fields] OR "portable"[All Fields] OR "portables"[All Fields])) OR ("Mobile Applications"[MeSH Terms] OR ("mobile"[All Fields] AND "applications"[All Fields]) OR "Mobile Applications"[All Fields] OR ("portable"[All Fields] AND "electronic"[All Fields] AND "app"[All Fields])) OR "Portable Electronic Applications"[All Fields] OR ("Mobile Applications"[MeSH Terms] OR ("mobile"[All Fields] AND "applications"[All Fields]) OR "Mobile Applications"[All Fields] OR ("application"[All Fields] AND "portable"[All Fields] AND "electronic"[All Fields])) OR "applications portable electronic"[All Fields] OR "electronic application portable"[All Fields] OR (("electronical"[All Fields] OR "electronically"[All Fields] OR "electronics"[MeSH Terms] OR "electronics"[All Fields] OR "electronic"[All Fields]) AND ("applicabilities"[All Fields] OR "applicability"[All Fields] OR "application"[All Fields] OR "applications"[All Fields] OR "applicative"[All Fields]) AND ("portability"[All Fields] OR "portable"[All Fields] OR "portables"[All Fields])) OR "Portable Electronic Application"[All Fields] OR ("Mobile Applications"[MeSH Terms] OR ("mobile"[All Fields] AND "applications"[All Fields]) OR "Mobile Applications"[All Fields] OR ("portable"[All Fields] AND "software"[All Fields] AND "apps"[All Fields])) OR ("Mobile Applications"[MeSH Terms] OR ("mobile"[All Fields] AND "applications"[All Fields]) OR "Mobile Applications"[All Fields] OR ("app"[All Fields] AND "portable"[All Fields] AND "software"[All Fields])) OR (("appl plant sci"[Journal] OR "apps"[All Fields]) AND ("portability"[All Fields] OR "portable"[All Fields] OR "portables"[All Fields]) AND ("software"[MeSH Terms] OR "software"[All Fields] OR "software s"[All Fields] OR "softwares"[All Fields])) OR ("Mobile Applications"[MeSH Terms] OR ("mobile"[All Fields] AND "applications"[All Fields]) OR "Mobile Applications"[All Fields] OR ("portable"[All Fields] AND "software"[All Fields] AND "app"[All Fields])) OR ("Mobile Applications"[MeSH Terms] OR ("mobile"[All Fields] AND "applications"[All Fields]) OR "Mobile Applications"[All Fields] OR ("software"[All Fields] AND "app"[All Fields] AND "portable"[All Fields])) OR (("software"[MeSH Terms] OR "software"[All Fields] OR "software s"[All Fields] OR "softwares"[All Fields]) AND ("appl plant sci"[Journal] OR "apps"[All Fields]) AND ("portability"[All Fields] OR "portable"[All Fields] OR "portables"[All Fields])) OR ("Mobile Applications"[MeSH Terms] OR ("mobile"[All Fields] AND "applications"[All Fields]) OR "Mobile Applications"[All Fields] OR ("portable"[All Fields] AND "software"[All Fields] AND "applications"[All Fields])) OR "application portable software"[All Fields] OR (("applicabilities"[All Fields] OR "applicability"[All Fields] OR "application"[All Fields] OR "applications"[All Fields] OR "applicative"[All Fields]) AND ("portability"[All Fields] OR "portable"[All Fields] OR "portables"[All Fields]) AND ("software"[MeSH Terms] OR "software"[All Fields] OR "software s"[All Fields] OR "softwares"[All Fields])) OR "Portable Software Application"[All Fields] OR ("Mobile Applications"[MeSH Terms] OR ("mobile"[All Fields] AND "applications"[All Fields]) OR "Mobile Applications"[All Fields] OR ("software"[All Fields] AND "application"[All Fields] AND "portable"[All Fields])) OR (("software"[MeSH Terms] OR "software"[All Fields] OR "software s"[All Fields] OR "softwares"[All Fields]) AND ("applicabilities"[All Fields] OR "applicability"[All Fields] OR "application"[All Fields] OR "applications"[All Fields] OR "applicative"[All Fields]) AND ("portability"[All Fields] OR "portable"[All Fields] OR "portables"[All Fields])) OR "Computer Software"[All Fields] OR "software computer"[All Fields] OR "Computer Programs"[All Fields] OR "Computer Program"[All Fields] OR "program computer"[All Fields] OR "programs computer"[All Fields] OR "Software Tools"[All Fields] OR "Software Tool"[All Fields] OR "tool software"[All Fields] OR "tools software"[All Fields] OR "Computer Applications Software"[All Fields] OR ("software"[MeSH Terms] OR "software"[All Fields] OR ("applications"[All Fields] AND "software"[All Fields] AND "Computer"[All Fields])) OR ("software"[MeSH Terms] OR "software"[All Fields] OR ("applications"[All Fields] AND "softwares"[All Fields] AND "Computer"[All Fields])) OR ("software"[MeSH Terms] OR "software"[All Fields] OR ("Computer"[All Fields] AND "applications"[All Fields] AND "softwares"[All Fields])) OR ("software"[MeSH Terms] OR "software"[All Fields] OR ("software"[All Fields] AND "Computer"[All Fields] AND "applications"[All Fields])) OR ("software"[MeSH Terms] OR "software"[All Fields] OR ("softwares"[All Fields] AND "Computer"[All Fields] AND "applications"[All Fields])) OR "Computer Software Applications"[All Fields] OR ("software"[MeSH Terms] OR "software"[All Fields] OR ("application"[All Fields] AND "Computer"[All Fields] AND "software"[All Fields])) OR "applications computer software"[All Fields] OR "Computer Software Application"[All Fields] OR "software application computer"[All Fields] OR "software applications computer"[All Fields] OR "applications medical informatics"[All Fields] OR ("medical informatics applications"[MeSH Terms] OR ("medical"[All Fields] AND "informatics"[All Fields] AND "applications"[All Fields]) OR "medical informatics applications"[All Fields] OR ("informatics"[All Fields] AND "applications"[All Fields] AND "medical"[All Fields])) OR ("medical informatics applications"[MeSH Terms] OR ("medical"[All Fields] AND "informatics"[All Fields] AND "applications"[All Fields]) OR "medical informatics applications"[All Fields] OR ("application"[All Fields] AND "medical"[All Fields] AND "informatics"[All Fields])) OR ("medical informatics applications"[MeSH Terms] OR ("medical"[All Fields] AND "informatics"[All Fields] AND "applications"[All Fields]) OR "medical informatics applications"[All Fields] OR ("informatics"[All Fields] AND "application"[All Fields] AND "medical"[All Fields])) OR "Medical Informatics Application"[All Fields] OR "Computer Programs and Programming"[All Fields] OR "Software Engineering"[All Fields] OR "engineering software"[All Fields] OR "virtual consultation"[All Fields] OR "virtual consultations"[All Fields] OR (("virtual"[All Fields] OR "virtuality"[All Fields] OR "virtualization"[All Fields] OR "virtualized"[All Fields] OR "virtualizing"[All Fields] OR "virtuals"[All Fields]) AND "online"[All Fields] AND ("consultancies"[All Fields] OR "consultancy"[All Fields] OR "consultant s"[All Fields] OR "consultants"[MeSH Terms] OR "consultants"[All Fields] OR "consultant"[All Fields] OR "consultative"[All Fields] OR "consulter"[All Fields] OR "consulters"[All Fields] OR "referral and consultation"[MeSH Terms] OR ("referral"[All Fields] AND "consultation"[All Fields]) OR "referral and consultation"[All Fields] OR "consult"[All Fields] OR "consultation"[All Fields] OR "consultations"[All Fields] OR "consulted"[All Fields] OR "consulting"[All Fields] OR "consults"[All Fields])) OR (("virtual"[All Fields] OR "virtuality"[All Fields] OR "virtualization"[All Fields] OR "virtualized"[All Fields] OR "virtualizing"[All Fields] OR "virtuals"[All Fields]) AND "online"[All Fields] AND ("consultancies"[All Fields] OR "consultancy"[All Fields] OR "consultant s"[All Fields] OR "consultants"[MeSH Terms] OR "consultants"[All Fields] OR "consultant"[All Fields] OR "consultative"[All Fields] OR "consulter"[All Fields] OR "consulters"[All Fields] OR "referral and consultation"[MeSH Terms] OR ("referral"[All Fields] AND "consultation"[All Fields]) OR "referral and consultation"[All Fields] OR "consult"[All Fields] OR "consultation"[All Fields] OR "consultations"[All Fields] OR "consulted"[All Fields] OR "consulting"[All Fields] OR "consults"[All Fields])) OR "Virtual Care"[All Fields] OR "E-consultation"[All Fields] OR "e-referral"[All Fields] OR "remote consultation"[All Fields] OR "tele-consultation"[All Fields] OR "video-consultation"[All Fields])  #3 - "neoplasms"[MeSH Terms] OR "neoplasms"[All Fields] OR "neoplasia"[All Fields] OR "neoplasias"[All Fields] OR "neoplasms"[MeSH Terms] OR "neoplasms"[All Fields] OR "neoplasia"[All Fields] OR "neoplasias"[All Fields] OR "neoplasm s"[All Fields] OR "neoplasms"[MeSH Terms] OR "neoplasms"[All Fields] OR "neoplasm"[All Fields] OR "cysts"[MeSH Terms] OR "cysts"[All Fields] OR "cyst"[All Fields] OR "neurofibroma"[MeSH Terms] OR "neurofibroma"[All Fields] OR "neurofibromas"[All Fields] OR "tumor s"[All Fields] OR "tumoral"[All Fields] OR "tumorous"[All Fields] OR "tumour"[All Fields] OR "neoplasms"[MeSH Terms] OR "neoplasms"[All Fields] OR "tumor"[All Fields] OR "tumour s"[All Fields] OR "tumoural"[All Fields] OR "tumourous"[All Fields] OR "tumours"[All Fields] OR "tumors"[All Fields] OR "cysts"[MeSH Terms] OR "cysts"[All Fields] OR "cyst"[All Fields] OR "neurofibroma"[MeSH Terms] OR "neurofibroma"[All Fields] OR "neurofibromas"[All Fields] OR "tumor s"[All Fields] OR "tumoral"[All Fields] OR "tumorous"[All Fields] OR "tumour"[All Fields] OR "neoplasms"[MeSH Terms] OR "neoplasms"[All Fields] OR "tumor"[All Fields] OR "tumour s"[All Fields] OR "tumoural"[All Fields] OR "tumourous"[All Fields] OR "tumours"[All Fields] OR "tumors"[All Fields] OR "cancer s"[All Fields] OR "cancerated"[All Fields] OR "canceration"[All Fields] OR "cancerization"[All Fields] OR "cancerized"[All Fields] OR "cancerous"[All Fields] OR "neoplasms"[MeSH Terms] OR "neoplasms"[All Fields] OR "cancer"[All Fields] OR "cancers"[All Fields] OR "cancer s"[All Fields] OR "cancerated"[All Fields] OR "canceration"[All Fields] OR "cancerization"[All Fields] OR "cancerized"[All Fields] OR "cancerous"[All Fields] OR "neoplasms"[MeSH Terms] OR "neoplasms"[All Fields] OR "cancer"[All Fields] OR "cancers"[All Fields] OR "malign"[All Fields] OR "malignance"[All Fields] OR "malignances"[All Fields] OR "malignant"[All Fields] OR "malignants"[All Fields] OR "malignities"[All Fields] OR "malignity"[All Fields] OR "malignization"[All Fields] OR "malignized"[All Fields] OR "maligns"[All Fields] OR "neoplasms"[MeSH Terms] OR "neoplasms"[All Fields] OR "malignancies"[All Fields] OR "malignancy"[All Fields] OR "malign"[All Fields] OR "malignance"[All Fields] OR "malignances"[All Fields] OR "malignant"[All Fields] OR "malignants"[All Fields] OR "malignities"[All Fields] OR "malignity"[All Fields] OR "malignization"[All Fields] OR "malignized"[All Fields] OR "maligns"[All Fields] OR "neoplasms"[MeSH Terms] OR "neoplasms"[All Fields] OR "malignancies"[All Fields] OR "malignancy"[All Fields] OR "Malignant Neoplasms"[All Fields] OR "Malignant Neoplasm"[All Fields] OR "neoplasm malignant"[All Fields] OR "neoplasms malignant"[All Fields] OR "Benign Neoplasms"[All Fields] OR "neoplasms benign"[All Fields] OR "Benign Neoplasm"[All Fields] OR "neoplasm benign"[All Fields]  #4 - "Systematic Review"[All Fields] OR "meta analysis"[All Fields] OR "Metanalysis"[All Fields] OR "meta analysis"[All Fields]  #5 - #1 AND #2 AND #3 AND #4 | **227** |
| **Cochrane Library** | #1 - diagnoses OR diagnose OR "Diagnoses and Examinations" OR "Examinations and Diagnoses" OR "Postmortem Diagnosis" OR "Diagnoses, Postmortem" OR "Diagnosis, Postmortem" OR "Postmortem Diagnoses" OR "Antemortem Diagnosis" OR "Antemortem Diagnoses" OR "Diagnoses, Antemortem" OR "Diagnosis, Antemortem" OR "Oral cancer diagnosis" OR "computer-aided diagnosis" OR " Cancer Early Detection" OR "Cancer Screening" OR "Screening, Cancer" OR "non-invasive screening" OR "Cancer Screening Tests" OR "Cancer Screening Test" OR "Screening Test, Cancer" OR "Screening Tests, Cancer" OR "Test, Cancer Screening" OR "Tests, Cancer Screening" OR "Early Diagnosis of Cancer" OR "Cancer Early Diagnosis"  #2 - ((Algorithm OR Algorithms OR "Intelligence, Artificial" OR "Computational Intelligence" OR "Intelligence, Computational" OR "Machine Intelligence" OR "Intelligence, Machine" OR "Computer Reasoning" OR "Reasoning, Computer" OR "AI (Artificial Intelligence)" OR “computer-assisted diagnosis (CAD)” OR “(CAD)” OR “Diagnosis, Computer Assisted” OR “Computer-Assisted Diagnosis” OR “Computer Assisted Diagnosis” OR “Computer-Assisted Diagnoses” OR “Diagnoses, Computer-Assisted” OR "Computer Vision Systems" OR "Computer Vision System" OR "System, Computer Vision" OR "Systems, Computer Vision" OR "Vision System, Computer" OR "Vision Systems, Computer" OR "Knowledge Acquisition (Computer)" OR "Acquisition, Knowledge (Computer)" OR "Knowledge Representation (Computer)" OR "Knowledge Representations (Computer)" OR "Representation, Knowledge (Computer)" OR "Computer Neural Network" OR "Computer Neural Networks" OR "Network, Computer Neural" OR "Networks, Computer Neural" OR "Neural Network, Computer" OR "Models, Neural Network" OR "Model, Neural Network" OR "Network Model, Neural" OR "Network Models, Neural" OR "Neural Network Model" OR "Neural Network Models" OR "Computational Neural Networks" OR "Computational Neural Network" OR "Network, Computational Neural" OR "Networks, Computational Neural" OR "Neural Network, Computational" OR "Neural Networks, Computational" OR "artificial neural network" OR "convolutional neural network" OR "Deep Learning" OR "Learning, Deep" OR "Hierarchical Learning" OR "Learning, Hierarchical" OR "Machine Learning" OR "Learning, Machine" OR "Transfer Learning" OR "Learning, Transfer") AND ( "asynchronous service models" OR "remote technologies" OR "remote technology" OR "virtual platform" OR "virtual platforms" OR "Mobile-phone-based screening" OR "Mobile Applications" OR "Application, Mobile" OR "Applications, Mobile" OR "Mobile Application" OR "Mobile Apps" OR "App, Mobile" OR "Apps, Mobile" OR "Mobile App" OR "Portable Electronic Apps" OR "App, Portable Electronic" OR "Apps, Portable Electronic" OR "Electronic App, Portable" OR "Electronic Apps, Portable" OR "Portable Electronic App" OR "Portable Electronic Applications" OR "Application, Portable Electronic" OR "Applications, Portable Electronic" OR "Electronic Application, Portable" OR "Electronic Applications, Portable" OR "Portable Electronic Application" OR "Portable Software Apps" OR "App, Portable Software" OR "Apps, Portable Software" OR "Portable Software App" OR "Software App, Portable" OR "Software Apps, Portable" OR "Portable Software Applications" OR "Application, Portable Software" OR "Applications, Portable Software" OR "Portable Software Application" OR "Software Application, Portable" OR "Software Applications, Portable" OR "Computer Software" OR "Software, Computer" OR "Computer Programs" OR "Computer Program" OR "Program, Computer" OR "Programs, Computer" OR "Software Tools" OR "Software Tool" OR "Tool, Software" OR "Tools, Software" OR "Computer Applications Software" OR "Applications Software, Computer" OR "Applications Softwares, Computer" OR "Computer Applications Softwares" OR "Software, Computer Applications" OR "Softwares, Computer Applications" OR "Computer Software Applications" OR "Application, Computer Software" OR "Applications, Computer Software" OR "Computer Software Application" OR "Software Application, Computer" OR "Software Applications, Computer" OR "Applications, Medical Informatics" OR "Informatics Applications, Medical" OR "Application, Medical Informatics" OR "Informatics Application, Medical" OR "Medical Informatics Application" OR "Computer Programs and Programming" OR "Software Engineering" OR "Engineering, Software" OR "virtual consultation" OR "virtual consultations" OR "Virtual online consultation" OR "Virtual online consultations" OR "Virtual Care" OR "E-consultation" OR "e-referral" OR "remote consultation" OR tele-consultation OR "video-consultation"))  #3 - Neoplasia OR Neoplasias OR Neoplasm OR Tumors OR Tumor OR Cancer OR Cancers OR Malignancy OR Malignancies OR "Malignant Neoplasms" OR "Malignant Neoplasm" OR "Neoplasm, Malignant" OR "Neoplasms, Malignant" OR "Benign Neoplasms" OR "Neoplasms, Benign" OR "Benign Neoplasm" OR "Neoplasm, Benign"  #4 "Systematic Review" OR "Meta-analysis" OR "Metanalysis" OR "Meta analysis"  #5 #1 AND #2 AND #3 AND #4 | **57** |
| **Scopus** | TITLE-ABS-KEY ( diagnoses OR diagnose OR "Diagnoses and Examinations" OR "Examinations and Diagnoses" OR "Postmortem Diagnosis" OR "Diagnoses, Postmortem" OR "Diagnosis, Postmortem" OR "Postmortem Diagnoses" OR "Antemortem Diagnosis" OR "Antemortem Diagnoses" OR "Diagnoses, Antemortem" OR "Diagnosis, Antemortem" OR "Oral cancer diagnosis" OR "computer-aided diagnosis" OR " Cancer Early Detection" OR "Cancer Screening" OR "Screening, Cancer" OR "non-invasive screening" OR "Cancer Screening Tests" OR "Cancer Screening Test" OR "Screening Test, Cancer" OR "Screening Tests, Cancer" OR "Test, Cancer Screening" OR "Tests, Cancer Screening" OR "Early Diagnosis of Cancer" OR "Cancer Early Diagnosis" ) AND TITLE-ABS-KEY ( ( algorithm OR algorithms OR "Intelligence, Artificial" OR "Computational Intelligence" OR "Intelligence, Computational" OR "Machine Intelligence" OR "Intelligence, Machine" OR "Computer Reasoning" OR "Reasoning, Computer" OR "AI (Artificial Intelligence)" OR "computer-assisted diagnosis (CAD)" OR "(CAD)" OR "Diagnosis, Computer Assisted" OR "Computer-Assisted Diagnosis" OR "Computer Assisted Diagnosis" OR "Computer-Assisted Diagnoses" OR "Diagnoses, Computer-Assisted" OR "Computer Vision Systems" OR "Computer Vision System" OR "System, Computer Vision" OR "Systems, Computer Vision" OR "Vision System, Computer" OR "Vision Systems, Computer" OR "Knowledge Acquisition (Computer)" OR "Acquisition, Knowledge (Computer)" OR "Knowledge Representation (Computer)" OR "Knowledge Representations (Computer)" OR "Representation, Knowledge (Computer)" OR "Computer Neural Network" OR "Computer Neural Networks" OR "Network, Computer Neural" OR "Networks, Computer Neural" OR "Neural Network, Computer" OR "Models, Neural Network" OR "Model, Neural Network" OR "Network Model, Neural" OR "Network Models, Neural" OR "Neural Network Model" OR "Neural Network Models" OR "Computational Neural Networks" OR "Computational Neural Network" OR "Network, Computational Neural" OR "Networks, Computational Neural" OR "Neural Network, Computational" OR "Neural Networks, Computational" OR "artificial neural network" OR "convolutional neural network" OR "Deep Learning" OR "Learning, Deep" OR "Hierarchical Learning" OR "Learning, Hierarchical" OR "Machine Learning" OR "Learning, Machine" OR "Transfer Learning" OR "Learning, Transfer" ) AND ( "asynchronous service models" OR "remote technologies" OR "remote technology" OR "virtual platform" OR "virtual platforms" OR "Mobile-phone-based screening" OR "Mobile Applications" OR "Application, Mobile" OR "Applications, Mobile" OR "Mobile Application" OR "Mobile Apps" OR "App, Mobile" OR "Apps, Mobile" OR "Mobile App" OR "Portable Electronic Apps" OR "App, Portable Electronic" OR "Apps, Portable Electronic" OR "Electronic App, Portable" OR "Electronic Apps, Portable" OR "Portable Electronic App" OR "Portable Electronic Applications" OR "Application, Portable Electronic" OR "Applications, Portable Electronic" OR "Electronic Application, Portable" OR "Electronic Applications, Portable" OR "Portable Electronic Application" OR "Portable Software Apps" OR "App, Portable Software" OR "Apps, Portable Software" OR "Portable Software App" OR "Software App, Portable" OR "Software Apps, Portable" OR "Portable Software Applications" OR "Application, Portable Software" OR "Applications, Portable Software" OR "Portable Software Application" OR "Software Application, Portable" OR "Software Applications, Portable" OR "Computer Software" OR "Software, Computer" OR "Computer Programs" OR "Computer Program" OR "Program, Computer" OR "Programs, Computer" OR "Software Tools" OR "Software Tool" OR "Tool, Software" OR "Tools, Software" OR "Computer Applications Software" OR "Applications Software, Computer" OR "Applications Softwares, Computer" OR "Computer Applications Softwares" OR "Software, Computer Applications" OR "Softwares, Computer Applications" OR "Computer Software Applications" OR "Application, Computer Software" OR "Applications, Computer Software" OR "Computer Software Application" OR "Software Application, Computer" OR "Software Applications, Computer" OR "Applications, Medical Informatics" OR "Informatics Applications, Medical" OR "Application, Medical Informatics" OR "Informatics Application, Medical" OR "Medical Informatics Application" OR "Computer Programs and Programming" OR "Software Engineering" OR "Engineering, Software" OR "virtual consultation" OR "virtual consultations" OR "Virtual online consultation" OR "Virtual online consultations" OR "Virtual Care" OR "E-consultation" OR "e-referral" OR "remote consultation" OR tele-consultation OR "video-consultation" ) ) TITLE-ABS-KEY ( neoplasia OR neoplasias OR neoplasm OR tumors OR tumor OR cancer OR cancers OR malignancy OR malignancies OR "Malignant Neoplasms" OR "Malignant Neoplasm" OR "Neoplasm, Malignant" OR "Neoplasms, Malignant" OR "Benign Neoplasms" OR "Neoplasms, Benign" OR "Benign Neoplasm" OR "Neoplasm, Benign" ) AND TITLE-ABS-KEY ( "Systematic Review" OR "Meta-analysis" OR "Metanalysis" OR "Meta analysis" ) | **22** |
| **Web of Science** | #1- TS=(Diagnoses OR Diagnose OR "Diagnoses and Examinations" OR "Examinations and Diagnoses" OR "Postmortem Diagnosis" OR "Diagnoses, Postmortem" OR "Diagnosis, Postmortem" OR "Postmortem Diagnoses" OR "Antemortem Diagnosis" OR "Antemortem Diagnoses" OR "Diagnoses, Antemortem" OR "Diagnosis, Antemortem" OR "Oral cancer diagnosis" OR "computer-aided diagnosis" OR" Cancer Early Detection" OR "Cancer Screening" OR "Screening, Cancer" OR "non-invasive screening" OR "Cancer Screening Tests" OR "Cancer Screening Test" OR "Screening Test, Cancer" OR "Screening Tests, Cancer" OR "Test, Cancer Screening" OR "Tests, Cancer Screening" OR "Early Diagnosis of Cancer" OR "Cancer Early Diagnosis")  #2- TS=((Algorithm OR Algorithms OR "Intelligence, Artificial" OR "Computational Intelligence" OR "Intelligence, Computational" OR "Machine Intelligence" OR "Intelligence, Machine" OR "Computer Reasoning" OR "Reasoning, Computer" OR "AI (Artificial Intelligence)" OR “computer-assisted diagnosis (CAD)” OR “(CAD)” OR “Diagnosis, Computer Assisted” OR “Computer-Assisted Diagnosis” OR “Computer Assisted Diagnosis” OR “Computer-Assisted Diagnoses” OR “Diagnoses, Computer-Assisted” OR "Computer Vision Systems" OR "Computer Vision System" OR "System, Computer Vision" OR "Systems, Computer Vision" OR "Vision System, Computer" OR "Vision Systems, Computer" OR "Knowledge Acquisition (Computer)" OR "Acquisition, Knowledge (Computer)" OR "Knowledge Representation (Computer)" OR "Knowledge Representations (Computer)" OR "Representation, Knowledge (Computer)" OR "Computer Neural Network" OR "Computer Neural Networks" OR "Network, Computer Neural" OR "Networks, Computer Neural" OR "Neural Network, Computer" OR "Models, Neural Network" OR "Model, Neural Network" OR "Network Model, Neural" OR "Network Models, Neural" OR "Neural Network Model" OR "Neural Network Models" OR "Computational Neural Networks" OR "Computational Neural Network" OR "Network, Computational Neural" OR "Networks, Computational Neural" OR "Neural Network, Computational" OR "Neural Networks, Computational" OR "artificial neural network" OR "convolutional neural network" OR "Deep Learning" OR "Learning, Deep" OR "Hierarchical Learning" OR "Learning, Hierarchical" OR "Machine Learning" OR "Learning, Machine" OR "Transfer Learning" OR "Learning, Transfer") AND ("asynchronous service models" OR "remote technologies" OR "remote technology" OR "virtual platform" OR "virtual platforms" OR "Mobile-phone-based screening" OR "Mobile Applications" OR "Application, Mobile" OR "Applications, Mobile" OR "Mobile Application" OR "Mobile Apps" OR "App, Mobile" OR "Apps, Mobile" OR "Mobile App" OR "Portable Electronic Apps" OR "App, Portable Electronic" OR "Apps, Portable Electronic" OR "Electronic App, Portable" OR "Electronic Apps, Portable" OR "Portable Electronic App" OR "Portable Electronic Applications" OR "Application, Portable Electronic" OR "Applications, Portable Electronic" OR "Electronic Application, Portable" OR "Electronic Applications, Portable" OR "Portable Electronic Application" OR "Portable Software Apps" OR "App, Portable Software" OR "Apps, Portable Software" OR "Portable Software App" OR "Software App, Portable" OR "Software Apps, Portable" OR "Portable Software Applications" OR "Application, Portable Software" OR "Applications, Portable Software" OR "Portable Software Application" OR "Software Application, Portable" OR "Software Applications, Portable" OR "Computer Software" OR "Software, Computer" OR "Computer Programs" OR "Computer Program" OR "Program, Computer" OR "Programs, Computer" OR "Software Tools" OR "Software Tool" OR "Tool, Software" OR "Tools, Software" OR "Computer Applications Software" OR "Applications Software, Computer" OR "Applications Softwares, Computer" OR "Computer Applications Softwares" OR "Software, Computer Applications" OR "Softwares, Computer Applications" OR "Computer Software Applications" OR "Application, Computer Software" OR "Applications, Computer Software" OR "Computer Software Application" OR "Software Application, Computer" OR "Software Applications, Computer" OR "Applications, Medical Informatics" OR "Informatics Applications, Medical" OR "Application, Medical Informatics" OR "Informatics Application, Medical" OR "Medical Informatics Application" OR "Computer Programs and Programming" OR "Software Engineering" OR "Engineering, Software" OR "virtual consultation" OR "virtual consultations" OR "Virtual online consultation" OR "Virtual online consultations" OR "Virtual Care" OR "E-consultation" OR "e-referral" OR "remote consultation" OR tele-consultation OR "video-consultation"))  #3– TS=(Neoplasia OR Neoplasias OR Neoplasm OR Tumors OR Tumor OR Cancer OR Cancers OR Malignancy OR Malignancies OR "Malignant Neoplasms" OR "Malignant Neoplasm" OR "Neoplasm, Malignant" OR "Neoplasms, Malignant" OR "Benign Neoplasms" OR "Neoplasms, Benign" OR "Benign Neoplasm" OR "Neoplasm, Benign")  #4 - TS=("Systematic Review" OR "Meta-analysis" OR "Metanalysis" OR "Meta analysis")  #5 - #4 AND #3 AND #2 AND #1 | **1** |
| **LILACS (via VHL Regional Portal)** | (Diagnoses OR Diagnose OR "Diagnoses and Examinations" OR "Examinations and Diagnoses" OR "Postmortem Diagnosis" OR "Diagnoses, Postmortem" OR "Diagnosis, Postmortem" OR "Postmortem Diagnoses" OR "Antemortem Diagnosis" OR "Antemortem Diagnoses" OR "Diagnoses, Antemortem" OR "Diagnosis, Antemortem" OR "Oral cancer diagnosis" OR "computer-aided diagnosis" OR" Cancer Early Detection" OR "Cancer Screening" OR "Screening, Cancer" OR "non-invasive screening" OR "Cancer Screening Tests" OR "Cancer Screening Test" OR "Screening Test, Cancer" OR "Screening Tests, Cancer" OR "Test, Cancer Screening" OR "Tests, Cancer Screening" OR "Early Diagnosis of Cancer" OR "Cancer Early Diagnosis") AND (((Algorithm OR Algorithms OR "Intelligence, Artificial" OR "Computational Intelligence" OR "Intelligence, Computational" OR "Machine Intelligence" OR "Intelligence, Machine" OR "Computer Reasoning" OR "Reasoning, Computer" OR "AI (Artificial Intelligence)" OR “computer-assisted diagnosis (CAD)” OR “(CAD)” OR “Diagnosis, Computer Assisted” OR “Computer-Assisted Diagnosis” OR “Computer Assisted Diagnosis” OR “Computer-Assisted Diagnoses” OR “Diagnoses, Computer-Assisted” OR "Computer Vision Systems" OR "Computer Vision System" OR "System, Computer Vision" OR "Systems, Computer Vision" OR "Vision System, Computer" OR "Vision Systems, Computer" OR "Knowledge Acquisition (Computer)" OR "Acquisition, Knowledge (Computer)" OR "Knowledge Representation (Computer)" OR "Knowledge Representations (Computer)" OR "Representation, Knowledge (Computer)" OR "Computer Neural Network" OR "Computer Neural Networks" OR "Network, Computer Neural" OR "Networks, Computer Neural" OR "Neural Network, Computer" OR "Models, Neural Network" OR "Model, Neural Network" OR "Network Model, Neural" OR "Network Models, Neural" OR "Neural Network Model" OR "Neural Network Models" OR "Computational Neural Networks" OR "Computational Neural Network" OR "Network, Computational Neural" OR "Networks, Computational Neural" OR "Neural Network, Computational" OR "Neural Networks, Computational" OR "artificial neural network" OR "convolutional neural network" OR "Deep Learning" OR "Learning, Deep" OR "Hierarchical Learning" OR "Learning, Hierarchical" OR "Machine Learning" OR "Learning, Machine" OR "Transfer Learning" OR "Learning, Transfer") AND ("asynchronous service models" OR "remote technologies" OR "remote technology" OR "virtual platform" OR "virtual platforms" OR "Mobile-phone-based screening" OR "Mobile Applications" OR "Application, Mobile" OR "Applications, Mobile" OR "Mobile Application" OR "Mobile Apps" OR "App, Mobile" OR "Apps, Mobile" OR "Mobile App" OR "Portable Electronic Apps" OR "App, Portable Electronic" OR "Apps, Portable Electronic" OR "Electronic App, Portable" OR "Electronic Apps, Portable" OR "Portable Electronic App" OR "Portable Electronic Applications" OR "Application, Portable Electronic" OR "Applications, Portable Electronic" OR "Electronic Application, Portable" OR "Electronic Applications, Portable" OR "Portable Electronic Application" OR "Portable Software Apps" OR "App, Portable Software" OR "Apps, Portable Software" OR "Portable Software App" OR "Software App, Portable" OR "Software Apps, Portable" OR "Portable Software Applications" OR "Application, Portable Software" OR "Applications, Portable Software" OR "Portable Software Application" OR "Software Application, Portable" OR "Software Applications, Portable" OR "Computer Software" OR "Software, Computer" OR "Computer Programs" OR "Computer Program" OR "Program, Computer" OR "Programs, Computer" OR "Software Tools" OR "Software Tool" OR "Tool, Software" OR "Tools, Software" OR "Computer Applications Software" OR "Applications Software, Computer" OR "Applications Softwares, Computer" OR "Computer Applications Softwares" OR "Software, Computer Applications" OR "Softwares, Computer Applications" OR "Computer Software Applications" OR "Application, Computer Software" OR "Applications, Computer Software" OR "Computer Software Application" OR "Software Application, Computer" OR "Software Applications, Computer" OR "Applications, Medical Informatics" OR "Informatics Applications, Medical" OR "Application, Medical Informatics" OR "Informatics Application, Medical" OR "Medical Informatics Application" OR "Computer Programs and Programming" OR "Software Engineering" OR "Engineering, Software" OR "virtual consultation" OR "virtual consultations" OR "Virtual online consultation" OR "Virtual online consultations" OR "Virtual Care" OR "E-consultation" OR "e-referral" OR "remote consultation" OR tele-consultation OR "video-consultation"))) AND (Neoplasia OR Neoplasias OR Neoplasm OR Tumors OR Tumor OR Cancer OR Cancers OR Malignancy OR Malignancies OR "Malignant Neoplasms" OR "Malignant Neoplasm" OR "Neoplasm, Malignant" OR "Neoplasms, Malignant" OR "Benign Neoplasms" OR "Neoplasms, Benign" OR "Benign Neoplasm" OR "Neoplasm, Benign") AND ("Systematic Review" OR "Meta-analysis" OR "Metanalysis" OR "Meta analysis") | **0** |
| **EMBASE** | ((diagnoses OR diagnose OR 'diagnoses and examinations' OR 'examinations and diagnoses'/exp OR 'examinations and diagnoses' OR 'postmortem diagnosis' OR 'diagnoses, postmortem' OR 'diagnosis, postmortem' OR 'postmortem diagnoses' OR 'antemortem diagnosis' OR 'antemortem diagnoses' OR 'diagnoses, antemortem' OR 'diagnosis, antemortem' OR 'oral cancer diagnosis' OR 'computer-aided diagnosis' OR 'cancer early detection' OR 'cancer screening'/exp OR 'cancer screening' OR 'screening, cancer'/exp OR 'screening, cancer' OR 'non-invasive screening' OR 'cancer screening tests' OR 'cancer screening test' OR 'screening test, cancer' OR 'screening tests, cancer' OR 'test, cancer screening' OR 'tests, cancer screening' OR 'early diagnosis of cancer' OR 'cancer early diagnosis') AND ('algorithm'/exp OR algorithm OR 'algorithms'/exp OR algorithms OR 'intelligence, artificial' OR 'computational intelligence'/exp OR 'computational intelligence' OR 'intelligence, computational' OR 'machine intelligence'/exp OR 'machine intelligence' OR 'intelligence, machine' OR 'computer reasoning'/exp OR 'computer reasoning' OR 'reasoning, computer' OR 'ai (artificial intelligence)' OR 'computer-assisted diagnosis (cad)' OR '(cad)' OR 'diagnosis, computer assisted'/exp OR 'diagnosis, computer assisted' OR 'computer-assisted diagnosis'/exp OR 'computer-assisted diagnosis' OR 'computer assisted diagnosis'/exp OR 'computer assisted diagnosis' OR 'computer-assisted diagnoses' OR 'diagnoses, computer-assisted' OR 'computer vision systems' OR 'computer vision system' OR 'system, computer vision' OR 'systems, computer vision' OR 'vision system, computer' OR 'vision systems, computer' OR 'knowledge acquisition (computer)' OR 'acquisition, knowledge (computer)' OR 'knowledge representation (computer)' OR 'knowledge representations (computer)' OR 'representation, knowledge (computer)' OR 'computer neural network'/exp OR 'computer neural network' OR 'computer neural networks'/exp OR 'computer neural networks' OR 'network, computer neural' OR 'networks, computer neural' OR 'neural network, computer'/exp OR 'neural network, computer' OR 'models, neural network' OR 'model, neural network' OR 'network model, neural' OR 'network models, neural' OR 'neural network model'/exp OR 'neural network model' OR 'neural network models' OR 'computational neural networks' OR 'computational neural network'/exp OR 'computational neural network' OR 'network, computational neural' OR 'networks, computational neural' OR 'neural network, computational' OR 'neural networks, computational' OR 'artificial neural network'/exp OR 'artificial neural network' OR 'convolutional neural network'/exp OR 'convolutional neural network' OR 'deep learning'/exp OR 'deep learning' OR 'learning, deep' OR 'hierarchical learning'/exp OR 'hierarchical learning' OR 'learning, hierarchical' OR 'machine learning'/exp OR 'machine learning' OR 'learning, machine'/exp OR 'learning, machine' OR 'transfer learning'/exp OR 'transfer learning' OR 'learning, transfer'/exp OR 'learning, transfer') AND ('asynchronous service models' OR 'remote technologies' OR 'remote technology' OR 'virtual platform' OR 'virtual platforms' OR 'mobile-phone-based screening' OR 'mobile applications'/exp OR 'mobile applications' OR 'application, mobile' OR 'applications, mobile' OR 'mobile application'/exp OR 'mobile application' OR 'mobile apps'/exp OR 'mobile apps' OR 'app, mobile' OR 'apps, mobile' OR 'mobile app'/exp OR 'mobile app' OR 'portable electronic apps' OR 'app, portable electronic' OR 'apps, portable electronic' OR 'electronic app, portable' OR 'electronic apps, portable' OR 'portable electronic app' OR 'portable electronic applications' OR 'application, portable electronic' OR 'applications, portable electronic' OR 'electronic application, portable' OR 'electronic applications, portable' OR 'portable electronic application' OR 'portable software apps'/exp OR 'portable software apps' OR 'app, portable software' OR 'apps, portable software' OR 'portable software app'/exp OR 'portable software app' OR 'software app, portable' OR 'software apps, portable' OR 'portable software applications'/exp OR 'portable software applications' OR 'application, portable software' OR 'applications, portable software' OR 'portable software application'/exp OR 'portable software application' OR 'software application, portable' OR 'software applications, portable' OR 'computer software' OR 'software, computer' OR 'computer programs' OR 'computer program'/exp OR 'computer program' OR 'program, computer'/exp OR 'program, computer' OR 'programs, computer' OR 'software tools' OR 'software tool' OR 'tool, software' OR 'tools, software' OR 'computer applications software' OR 'applications software, computer' OR 'applications softwares, computer' OR 'computer applications softwares' OR 'software, computer applications' OR 'softwares, computer applications' OR 'computer software applications' OR 'application, computer software' OR 'applications, computer software' OR 'computer software application' OR 'software application, computer' OR 'software applications, computer' OR 'applications, medical informatics' OR 'informatics applications, medical' OR 'application, medical informatics' OR 'informatics application, medical' OR 'medical informatics application' OR 'computer programs and programming'/exp OR 'computer programs and programming' OR 'software engineering' OR 'engineering, software' OR 'virtual consultation' OR 'virtual consultations' OR 'virtual online consultation' OR 'virtual online consultations' OR 'virtual care'/exp OR 'virtual care' OR 'e-consultation'/exp OR 'e-consultation' OR 'e-referral' OR 'remote consultation'/exp OR 'remote consultation' OR 'tele consultation'/exp OR 'tele consultation' OR 'video-consultation'/exp OR 'video-consultation') AND ('neoplasia'/exp OR neoplasia OR neoplasias OR 'neoplasm'/exp OR neoplasm OR 'tumors'/exp OR tumors OR 'tumor'/exp OR tumor OR 'cancer'/exp OR cancer OR 'cancers'/exp OR cancers OR 'malignancy'/exp OR malignancy OR 'malignancies'/exp OR malignancies OR 'malignant neoplasms' OR 'malignant neoplasm'/exp OR 'malignant neoplasm' OR 'neoplasm, malignant' OR 'neoplasms, malignant' OR 'benign neoplasms' OR 'neoplasms, benign' OR 'benign neoplasm'/exp OR 'benign neoplasm' OR 'neoplasm, benign'/exp OR 'neoplasm, benign') AND ('systematic review'/exp OR 'systematic review' OR 'meta-analysis'/exp OR 'meta-analysis' OR 'metanalysis' OR 'meta analysis'/exp OR 'meta analysis') | **30** |
| **EBSCOhost**  **(All databases)** | (diagnoses OR diagnose OR "Diagnoses and Examinations" OR "Examinations and Diagnoses" OR "Postmortem Diagnosis" OR "Diagnoses, Postmortem" OR "Diagnosis, Postmortem" OR "Postmortem Diagnoses" OR "Antemortem Diagnosis" OR "Antemortem Diagnoses" OR "Diagnoses, Antemortem" OR "Diagnosis, Antemortem" OR "Oral cancer diagnosis" OR "computer-aided diagnosis" OR " Cancer Early Detection" OR "Cancer Screening" OR "Screening, Cancer" OR "non-invasive screening" OR "Cancer Screening Tests" OR "Cancer Screening Test" OR "Screening Test, Cancer" OR "Screening Tests, Cancer" OR "Test, Cancer Screening" OR "Tests, Cancer Screening" OR "Early Diagnosis of Cancer" OR "Cancer Early Diagnosis" ) AND ( ((Algorithm OR Algorithms OR "Intelligence, Artificial" OR "Computational Intelligence" OR "Intelligence, Computational" OR "Machine Intelligence" OR "Intelligence, Machine" OR "Computer Reasoning" OR "Reasoning, Computer" OR "AI (Artificial Intelligence)" OR “computer-assisted diagnosis (CAD)” OR “(CAD)” OR “Diagnosis, Computer Assisted” OR “Computer-Assisted Diagnosis” OR “Computer Assisted Diagnosis” OR “Computer-Assisted Diagnoses” OR “Diagnoses, Computer-Assisted” OR "Computer Vision Systems" OR "Computer Vision System" OR "System, Computer Vision" OR "Systems, Computer Vision" OR "Vision System, Computer" OR "Vision Systems, Computer" OR "Knowledge Acquisition (Computer)" OR "Acquisition, Knowledge (Computer)" OR "Knowledge Representation (Computer)" OR "Knowledge Representations (Computer)" OR "Representation, Knowledge (Computer)" OR "Computer Neural Network" OR "Computer Neural Networks" OR "Network, Computer Neural" OR "Networks, Computer Neural" OR "Neural Network, Computer" OR "Models, Neural Network" OR "Model, Neural Network" OR "Network Model, Neural" OR "Network Models, Neural" OR "Neural Network Model" OR "Neural Network Models" OR "Computational Neural Networks" OR "Computational Neural Network" OR "Network, Computational Neural" OR "Networks, Computational Neural" OR "Neural Network, Computational" OR "Neural Networks, Computational" OR "artificial neural network" OR "convolutional neural network" OR "Deep Learning" OR "Learning, Deep" OR "Hierarchical Learning" OR "Learning, Hierarchical" OR "Machine Learning" OR "Learning, Machine" OR "Transfer Learning" OR "Learning, Transfer") AND ("asynchronous service models" OR "remote technologies" OR "remote technology" OR "virtual platform" OR "virtual platforms" OR "Mobile-phone-based screening" OR "Mobile Applications" OR "Application, Mobile" OR "Applications, Mobile" OR "Mobile Application" OR "Mobile Apps" OR "App, Mobile" OR "Apps, Mobile" OR "Mobile App" OR "Portable Electronic Apps" OR "App, Portable Electronic" OR "Apps, Portable Electronic" OR "Electronic App, Portable" OR "Electronic Apps, Portable" OR "Portable Electronic App" OR "Portable Electronic Applications" OR "Application, Portable Electronic" OR "Applications, Portable Electronic" OR "Electronic Application, Portable" OR "Electronic Applications, Portable" OR "Portable Electronic Application" OR "Portable Software Apps" OR "App, Portable Software" OR "Apps, Portable Software" OR "Portable Software App" OR "Software App, Portable" OR "Software Apps, Portable" OR "Portable Software Applications" OR "Application, Portable Software" OR "Applications, Portable Software" OR "Portable Software Application" OR "Software Application, Portable" OR "Software Applications, Portable" OR "Computer Software" OR "Software, Computer" OR "Computer Programs" OR "Computer Program" OR "Program, Computer" OR "Programs, Computer" OR "Software Tools" OR "Software Tool" OR "Tool, Software" OR "Tools, Software" OR "Computer Applications Software" OR "Applications Software, Computer" OR "Applications Softwares, Computer" OR "Computer Applications Softwares" OR "Software, Computer Applications" OR "Softwares, Computer Applications" OR "Computer Software Applications" OR "Application, Computer Software" OR "Applications, Computer Software" OR "Computer Software Application" OR "Software Application, Computer" OR "Software Applications, Computer" OR "Applications, Medical Informatics" OR "Informatics Applications, Medical" OR "Application, Medical Informatics" OR "Informatics Application, Medical" OR "Medical Informatics Application" OR "Computer Programs and Programming" OR "Software Engineering" OR "Engineering, Software" OR "virtual consultation" OR "virtual consultations" OR "Virtual online consultation" OR "Virtual online consultations" OR "Virtual Care" OR "E-consultation" OR "e-referral" OR "remote consultation" OR tele-consultation OR "video-consultation")) ) AND ( Neoplasia OR Neoplasias OR Neoplasm OR Tumors OR Tumor OR Cancer OR Cancers OR Malignancy OR Malignancies OR "Malignant Neoplasms" OR "Malignant Neoplasm" OR "Neoplasm, Malignant" OR "Neoplasms, Malignant" OR "Benign Neoplasms" OR "Neoplasms, Benign" OR "Benign Neoplasm" OR "Neoplasm, Benign" ) AND ( "Systematic Review" OR "Meta-analysis" OR "Metanalysis" OR "Meta analysis") | **5** |
| ***SCIELO*** | (Diagnoses OR Diagnose OR "Oral cancer diagnosis" OR "computer-aided diagnosis" OR "Cancer Early Detection" OR "Cancer Screening" OR "Cancer Screening Test" OR "Early Diagnosis of Cancer" OR "Cancer Early Diagnosis") AND ((Algorithm OR Algorithms OR "Intelligence, Artificial" OR "Computational Intelligence" OR "Machine Intelligence" OR "Computer Reasoning" OR "AI (Artificial Intelligence)" OR “computer-assisted diagnosis (CAD)” OR “(CAD)” OR “Diagnosis, Computer Assisted” OR “Computer-Assisted Diagnosis” OR “Computer Assisted Diagnosis” OR “Computer-Assisted Diagnoses” OR “Diagnoses, Computer-Assisted” OR "Computer Vision Systems" OR "Knowledge Acquisition (Computer)" OR "Computer Neural Network" OR "Computer Neural Networks" OR "Neural Network Model" OR "Neural Network Models" OR "Computational Neural Networks" OR "Computational Neural Network" OR "artificial neural network" OR "convolutional neural network" OR "Deep Learning" OR "Hierarchical Learning" OR "Machine Learning" OR "Transfer Learning") AND ("asynchronous service models" OR "remote technologies" OR "remote technology" OR "virtual platform" OR "virtual platforms" OR "Mobile-phone-based screening" OR "Mobile Applications" OR "Application, Mobile" OR "Applications, Mobile" OR "Mobile Application" OR "Mobile Apps" OR "Mobile App" OR "Portable Electronic Apps" OR "Portable Electronic App" OR "Portable Electronic Applications" OR "Portable Electronic Application" OR "Portable Software Apps" OR "Portable Software Applications" OR "Portable Software Application" OR "Computer Programs" OR "Computer Program" OR "Software Tools" OR "Software Tool" OR "Computer Applications Software" OR "Computer Applications Softwares" OR "Computer Software Applications" OR "Computer Software Application" OR "Medical Informatics Application" OR "Computer Programs and Programming" OR "Software Engineering" OR "virtual consultation" OR "virtual consultations" OR "Virtual online consultation" OR "Virtual online consultations" OR "Virtual Care" OR "E-consultation" OR "e-referral" OR "remote consultation" OR tele-consultation OR "video-consultation")) AND (Neoplasia OR Neoplasias OR Neoplasm OR Tumors OR Tumor OR Cancer OR Cancers OR Malignancy OR Malignancies OR "Malignant Neoplasms" OR "Malignant Neoplasm" OR "Benign Neoplasms" OR "Benign Neoplasm") AND ("Systematic Review" OR "Meta-analysis" OR "Metanalysis" OR "Meta analysis") | **0** |
| ***PROQUEST*** | noft(diagnoses OR diagnose OR "Diagnoses and Examinations" OR "Examinations and Diagnoses" OR "Postmortem Diagnosis" OR "Diagnoses, Postmortem" OR "Diagnosis, Postmortem" OR "Postmortem Diagnoses" OR "Antemortem Diagnosis" OR "Antemortem Diagnoses" OR "Diagnoses, Antemortem" OR "Diagnosis, Antemortem" OR "Oral cancer diagnosis" OR "computer-aided diagnosis" OR " Cancer Early Detection" OR "Cancer Screening" OR "Screening, Cancer" OR "non-invasive screening" OR "Cancer Screening Tests" OR "Cancer Screening Test" OR "Screening Test, Cancer" OR "Screening Tests, Cancer" OR "Test, Cancer Screening" OR "Tests, Cancer Screening" OR "Early Diagnosis of Cancer" OR "Cancer Early Diagnosis") AND noft(((Algorithm OR Algorithms OR "Intelligence, Artificial" OR "Computational Intelligence" OR "Intelligence, Computational" OR "Machine Intelligence" OR "Intelligence, Machine" OR "Computer Reasoning" OR "Reasoning, Computer" OR "AI (Artificial Intelligence)" OR "computer-assisted diagnosis (CAD)" OR "(CAD)" OR "Diagnosis, Computer Assisted" OR "Computer-Assisted Diagnosis" OR "Computer Assisted Diagnosis" OR "Computer-Assisted Diagnoses" OR "Diagnoses, Computer-Assisted" OR "Computer Vision Systems" OR "Computer Vision System" OR "System, Computer Vision" OR "Systems, Computer Vision" OR "Vision System, Computer" OR "Vision Systems, Computer" OR "Knowledge Acquisition (Computer)" OR "Acquisition, Knowledge (Computer)" OR "Knowledge Representation (Computer)" OR "Knowledge Representations (Computer)" OR "Representation, Knowledge (Computer)" OR "Computer Neural Network" OR "Computer Neural Networks" OR "Network, Computer Neural" OR "Networks, Computer Neural" OR "Neural Network, Computer" OR "Models, Neural Network" OR "Model, Neural Network" OR "Network Model, Neural" OR "Network Models, Neural" OR "Neural Network Model" OR "Neural Network Models" OR "Computational Neural Networks" OR "Computational Neural Network" OR "Network, Computational Neural" OR "Networks, Computational Neural" OR "Neural Network, Computational" OR "Neural Networks, Computational" OR "artificial neural network" OR "convolutional neural network" OR "Deep Learning" OR "Learning, Deep" OR "Hierarchical Learning" OR "Learning, Hierarchical" OR "Machine Learning" OR "Learning, Machine" OR "Transfer Learning" OR "Learning, Transfer") AND ("asynchronous service models" OR "remote technologies" OR "remote technology" OR "virtual platform" OR "virtual platforms" OR "Mobile-phone-based screening" OR "Mobile Applications" OR "Application, Mobile" OR "Applications, Mobile" OR "Mobile Application" OR "Mobile Apps" OR "App, Mobile" OR "Apps, Mobile" OR "Mobile App" OR "Portable Electronic Apps" OR "App, Portable Electronic" OR "Apps, Portable Electronic" OR "Electronic App, Portable" OR "Electronic Apps, Portable" OR "Portable Electronic App" OR "Portable Electronic Applications" OR "Application, Portable Electronic" OR "Applications, Portable Electronic" OR "Electronic Application, Portable" OR "Electronic Applications, Portable" OR "Portable Electronic Application" OR "Portable Software Apps" OR "App, Portable Software" OR "Apps, Portable Software" OR "Portable Software App" OR "Software App, Portable" OR "Software Apps, Portable" OR "Portable Software Applications" OR "Application, Portable Software" OR "Applications, Portable Software" OR "Portable Software Application" OR "Software Application, Portable" OR "Software Applications, Portable" OR "Computer Software" OR "Software, Computer" OR "Computer Programs" OR "Computer Program" OR "Program, Computer" OR "Programs, Computer" OR "Software Tools" OR "Software Tool" OR "Tool, Software" OR "Tools, Software" OR "Computer Applications Software" OR "Applications Software, Computer" OR "Applications Softwares, Computer" OR "Computer Applications Softwares" OR "Software, Computer Applications" OR "Softwares, Computer Applications" OR "Computer Software Applications" OR "Application, Computer Software" OR "Applications, Computer Software" OR "Computer Software Application" OR "Software Application, Computer" OR "Software Applications, Computer" OR "Applications, Medical Informatics" OR "Informatics Applications, Medical" OR "Application, Medical Informatics" OR "Informatics Application, Medical" OR "Medical Informatics Application" OR "Computer Programs and Programming" OR "Software Engineering" OR "Engineering, Software" OR "virtual consultation" OR "virtual consultations" OR "Virtual online consultation" OR "Virtual online consultations" OR "Virtual Care" OR "E-consultation" OR "e-referral" OR "remote consultation" OR tele-consultation OR "video-consultation"))) AND noft(Neoplasia OR Neoplasias OR Neoplasm OR Tumors OR Tumor OR Cancer OR Cancers OR Malignancy OR Malignancies OR "Malignant Neoplasms" OR "Malignant Neoplasm" OR "Neoplasm, Malignant" OR "Neoplasms, Malignant" OR "Benign Neoplasms" OR "Neoplasms, Benign" OR "Benign Neoplasm" OR "Neoplasm, Benign") AND noft("Systematic Review" OR "Meta-analysis" OR "Metanalysis" OR "Meta analysis") | **1** |
| **Google Scholar** | **With all words:** (Diagnose OR "Oral cancer diagnosis" OR "computer-aided diagnosis" OR "Cancer Early Detection") AND (("Intelligence, Artificial" OR "Machine Learning" OR "Deep Learning" OR "Neural Network Model" OR "convolutional neural network") AND ("remote technology" OR "virtual platform" OR "Mobile-phone-based screening" OR "Portable Software Application" OR "Mobile Application" OR "Mobile App" OR "Computer Software Application" OR "virtual consultation" OR "E-consultation" OR tele-consultation))  com a frase exata: "Systematic Review" OR "Meta-analysis" OR "Metanalysis" OR "Meta analysis"  com no mínimo uma das palavras: Neoplasia OR Neoplasm OR Tumor OR Cancer OR "Malignant Neoplasm" OR "Benign Neoplasm" | **39** |
| **JSTOR** | *("Cancer Early Detection" ) AND ("Artificial Intelligence ") AND ("Mobile Application" OR "Computer Software Application") AND (Neoplasia OR Neoplasm) AND ("Systematic Review")* | **0** |
