# Supplementary material for: The use of artificial intelligence tools in cancer detection compared to the traditional diagnostic imaging methods: An overview of the systematic reviews
Source: PLoS One. 2023 Oct 5;18(10):e0292063. doi: 10.1371/journal.pone.0292063 (PMC10553229; doi:10.1371/journal.pone.0292063)
Supplement: S2 Table — Legend—1—Studies evaluating diagnosis of areas other than medicine and dentistry (Physiotherapist, Nutritionist, Nurse, Caregivers etc.); 2 –Patients with a confirmed diagnosis of cancer; 3—Systematic Reviews not evaluating the diagnostic accuracy Artificial intelligence, Machine learning, Deep learning and Convolutional Neural Networks; 4—Systematic Reviews with Artificial intelligence use for other diseases diagnosis (Diabetes, Hypertension, etc); 5—Systematic reviews in which AI was not compared to a reference test; 6—Systematic reviews evaluating other technologies for early detection or cancer diagnosis (spectrometry, biomarkers, autofluorescence, Multispectral widefield optical imaging, optical instruments, robotic equipment etc.); 7—literature reviews, integrative reviews, narrative reviews, overviews; 8—Editorials∕Letters; 9—Conferences, Summaries, abstracts and posters; 10—In vitro studies; 11—Studies of animal models; 12—Thesis and Dissertations and book chapters; 13—Pipelines, guidelines and research protocols; 14—Review papers that do not follow the inclusion criteria adopted for the definition of Systematic Reviews; 15—Primary studies of any type; 16—No full paper avaliable. (DOC) [file pone.0292063.s003.doc]

S2 Table - Excluded articles and reasons for exclusion (n=23).

| Author, year | Reasons for exclusions |
| --- | --- |
| Bartosch-Härlid A. (1) 2008. | 14 |
| Nayantara PV. (2) 2020. | 14 |
| Herman RA . (3), 2012 | 14 |
| Simões PW . (4), 2015 | 06 |
| Kourou K . (5), 2021 | 07 |
| Drouin SJ. (6), 2013 | 10 |
| Gudigar A. (7), 2020 | 02 |
| Mahmood H. (8), 2020 | 11 |
| Alsalem MA. (9), 2018 | 06 |
| Abraha I. (10), 2018 | 02 |
| Abbod MF . (11), 2007 | 06 |
| Tian Y . (12), 2018 | 15 |
| Yassin NIR. (13), 2018 | 05 |
| Valente IR. (14), 2016 | 14 |
| Amir GJ. (15), 2019 | 14 |
| Robinson C. (16), 2008 | 14 |
| Staal FCR. (17), 2021 | 14 |
| Ursprung S. (18), 2020 | 3 |
| Rajgor AD. (19), 2021 | 16 |
| Badrigilan S. (21), 2021 | 3 |
| Limardo A. (14), 2021 | 16 |
| Taylor P. (16), | 14 |
| Noble, M. (17) | 14 |

1. 1 - Studies evaluating diagnosis of areas other than medicine and dentistry (Physiotherapist, Nutritionist, Nurse, Caregivers etc.); 2 – Patients with a confirmed diagnosis of cancer; 3 - Systematic Reviews not evaluating the diagnostic accuracy Artificial intelligence, Machine learning, Deep learning and Convolutional Neural Networks; 4 - Systematic Reviews with Artificial intelligence use for other diseases diagnosis (Diabetes, Hypertension, etc); 5 - Systematic reviews in which AI was not compared to a reference test; 6 - Systematic reviews evaluating other technologies for early detection or cancer diagnosis (spectrometry, biomarkers, autofluorescence, Multispectral widefield optical imaging, optical instruments, robotic equipment etc.); 7 - literature reviews, integrative reviews, narrative reviews, overviews; 8 - Editorials∕Letters; 9 - Conferences, Summaries, abstracts and posters; 10 - In vitro studies; 11 - Studies of animal models; 12 - Thesis and Dissertations and book chapters; 13 - Pipelines, guidelines and research protocols; 14 - Review papers that do not follow the inclusion criteria adopted for the definition of Systematic Reviews; 15 - Primary studies of any type; 16 - No full paper avaliable.

REFERENCES

1. Bartosch-Härlid A, Andersson B, Aho U, Nilsson J, Andersson R. Artificial neural networks in pancreatic disease. Br J Surg. 2008; 95(7): 817-826. DOI:10.1002/bjs.6239.
2. Nayantara PV, Kamath S, Manjunath KN, Rajagopal KV. Computer-aided diagnosis of liver lesions using CT images: A systematic review. Comput Biol Med. 2020; 127:104035. DOI: 10.1016/j.compbiomed.2020.104035.
3. Herman RA, Gilchrist B, Link BK, Carnahan R. A systematic review of validated methods for identifying lymphoma using administrative data. Pharmacoepidemiol Drug Saf. 2012; Suppl 1: 203-212. DOI: 10.1002/pds.2315.
4. Simões PW, Silva GD, Moretti GP, Simon CS, Winnikow EP, Nassar SM, et al. Meta analysis of the use of Bayesian networks in breast cancer diagnosis. Cad Saude Publica. 2015; 31(1): 26-38. DOI: 10.1590/0102-311x00205213.
5. Kourou K, Exarchos KP, Papaloukas C, Sakaloglou P, Exarchos T, Fotiadis DI. Applied machine learning in cancer research: A systematic review for patient diagnosis, classification and prognosis. Comput Struct Biotechnol J. 2021; 19: 5546-5555. DOI: 10.1016/j.csbj.2021.10.006.
6. Drouin SJ, Yates DR, Hupertan V, Cussenot O, Rouprêt M. A systematic review of the tools available for predicting survival and managing patients with urothelial carcinomas of the bladder and of the upper tract in a curative setting. World J Uro. 2013; 31(1): 109-116. DOI: 10.1007/s00345-012-1008-9.
7. Gudigar A, Raghavendra U, Hegde A, Kalyani M, Ciaccio EJ, Rajendra Acharya U. Brain pathology identification using computer aided diagnostic tool: A systematic review. Comput Methods Programs Biomed. 2020; 187: 105205. DOI: 10.1016/j.cmpb.2019.105205.
8. Mahmood H, Shaban M, Indave BI, Santos-Silva AR, Rajpoot N, Khurram SA. Use of artificial intelligence in diagnosis of head and neck precancerous and cancerous lesions: A systematic review. Oral Oncol. 2020; 110: 104885. DOI: 10.1016/j.oraloncology.2020.104885.
9. Alsalem MA, Zaidan AA, Zaidan BB, Hashim M, Madhloom HT, Azeez ND. A review of the automated detection and classification of acute leukaemia: Coherent taxonomy, datasets, validation and performance measurements, motivation, open challenges and recommendations. Comput Methods Programs Biomed. 2018; 158: 93-112 DOI: 10.1016/j.cmpb.2018.02.005.
10. Abraha I, Montedori A, Serraino D, Orso M, Giovannini G, Scotti V, et al. Accuracy of administrative databases in detecting primary breast cancer diagnoses: a systematic review. BMJ Open. 2018; 8(7): e019264. DOI: 10.1136/bmjopen-2017-019264.
11. Abbod MF, Catto JW, Linkens DA, Hamdy FC. Application of artificial intelligence to the management of urological cancer. J Urol. 2007; 178(4 Pt 1): 1150-1156. DOI: 10.1016/j.juro.2007.05.122.
12. Tian Y, Shang Y, Tong DY, Chi SQ, Li J, Kong XX, et al, POPCORN: A web service for individual PrognOsis prediction based on multi-center clinical data CollabORatioN without patient-level data sharing. J Biomed Inform. 2018; 86: 1-14. DOI: 10.1016/j.jbi.2018.08.008.
13. Yassin NIR, Omran S, El Houby EMF, Allam H. Machine learning techniques for breast cancer computer aided diagnosis using different image modalities: A systematic review. Comput Methods Programs Biomed. 2018; 156: 25-45.DOI: 10.1016/j.cmpb.2017.12.012.
14. Valente IR, Cortez PC, Neto EC, Soares JM, de Albuquerque VH, Tavares JM. Automatic 3D pulmonary nodule detection in CT images: A survey. Comput Methods Programs Biomed. 2016; 124: 91-107. DOI: 10.1016/j.cmpb.2015.10.006.
15. Amir GJ, Lehmann HP. After Detection: The Improved Accuracy of Lung Cancer Assessment Using Radiologic Computer-aided Diagnosis. Acad Radiol. 2016; 23(2): 186-191. DOI: 10.1016/j.acra.2015.10.014.
16. Robinson C, Halligan S, Taylor SA, Mallett S, Altman DG. CT colonography: a systematic review of standard of reporting for studies of computer-aided detection. Radiology. 2008; 246(2): 426-433. DOI: 10.1148/radiol.2461070121.
17. Staal FCR, van der Reijd DJ, Taghavi M, Lambregts DMJ, Beets-Tan RGH, Maas M. Radiomics for the Prediction of Treatment Outcome and Survival in Patients With Colorectal Cancer: A Systematic Review. Clin Colorectal Cancer. 2021; 20(1): 52-71. DOI: 10.1016/j.clcc.2020.11.001.
18. Ursprung S, Beer L, Bruining A, Woitek R, Stewart GD, Gallagher FA, et al. Radiomics of computed tomography and magnetic resonance imaging in renal cell carcinoma-a systematic review and meta-analysis. Eur Radiol. 2020; 30(6):3558-3566. DOI: 10.1007/s00330-020-06666-3.
19. Rajgor AD, Patel S, McCulloch D, Obara B, Bacardit J, McQueen A, et al. The application of radiomics in laryngeal cancer. Br J Radiol. 2021; 94(1128): 20210499. DOI: 10.1259/bjr.20210499.
20. Badrigilan S, Nabavi S, Abin AA, Rostampour N, Abedi I, Shirvani A, et al. Deep learning approaches for automated classification and segmentation of head and neck cancers and brain tumors in magnetic resonance images: a meta-analysis study. Int J Comput Assist Radiol Surg. 2021; 16(4): 529-542. DOI: 10.1007/s11548-021-02326-z.
21. Limardo A, Blanco L, Menendez J, García L, Ortega A. The development of a clinical algorithm for the diagnosis of tumours in the parapharyngeal space. A systematic review. Acta Otorrinolaringol Esp (Engl Ed). 2021; S0001-6519(20)30192-8. DOI: 10.1016/j.otorri.2020.11.001.
22. Taylor P, Potts HW. Computer aids and human second reading as interventions in screening mammography: two systematic reviews to compare effects on cancer detection and recall rate. Eur J Cancer. 2008; 44(6): 798-807. DOI: 10.1016/j.ejca.2008.02.016.
23. Noble M, Bruening W, Uhl S, Schoelles K. Computer-aided detection mammography for breast cancer screening: systematic review and meta-analysis. Arch Gynecol Obstet. 2009; 279(6): 881-890. DOI: 10.1007/s00404-008-0841-y.
