# Supplementary material for: The use of artificial intelligence tools in cancer detection compared to the traditional diagnostic imaging methods: An overview of the systematic reviews
Source: PLoS One. 2023 Oct 5;18(10):e0292063. doi: 10.1371/journal.pone.0292063 (PMC10553229; doi:10.1371/journal.pone.0292063)
Supplement: S3 Table — (DOCX) [file pone.0292063.s004.docx]

S3 Table - Over Laping (n=09).

| **Author,**  **year** | **Included Studies** | **1** | **2** | **3** | **4** | **5** | ***6*** | ***7*** | **8** | **9** | **10** | **11** | **12** | **13** | **14** | **15** | **16** | **17** | **18** |
| --- | --- | --- | --- | --- | --- | --- | --- | --- | --- | --- | --- | --- | --- | --- | --- | --- | --- | --- | --- |
| Dorrius et al, 2011, | 10 | Arazi-Kleinman 2009 | Meeuwis 2009 | Baltzer 2009 | Baltzer 2009 | Veltman [ 2009 | Renz 2008 | Hauth 2008 | Williams 2006 | Lehman 2005 | Kelcz 2002 |  |  |  |  |  |  |  |  |
| Nindrea et al, 2018, | 11 | Chang et al., 2003 | Polat and Gunes, 2007 | Heidari et al., 2018 | Ayer et al., 2010 | Dramicanin et al., 2012 | Subramanian et al., 2014 | Mert et al., 2015 | Milosevic et al., 2015 | Sun et al., 2015 | Asri et al., 2016 | Akay, 2009 |  |  |  |  |  |  |  |
| Eadie et al,  2012, | 48 | Andre M, et al 2007 | Awai K, et al 2006 | Ayer T, et al 2010 | Balleyguier C, et all 2005 | Brem RF, et al 2001 | Chang W-L, et al 2010 | Chen H, et al 2010 | Choi EJ, et al 2008 | Ciatto S,et al 2003 | Claridge E, et al 1992 | Fenton JJ, et al 2007 | Helm EJ, et al 2009 | Horsch K, et al 2006 | Horsch K, et al 2004 | Huo ZM, et al 2002 | Jesneck JL, et al 2007 | Jiang YL, et al 2006 | Juntu J, et al 2010 |
|  |  | Karahaliou AN, et al 2008 | Kegelmeyer WP, et al 1994 | Lauria A. et al 2009 | Lee IJ,, et al 2005 | Leichter I, et all 2000 | Leichter I, et al 2000 | Llobet R, et al 2007 | Lo JY, et al 1995 | Mani A, et al 2004 | Marx C, et al 2004 | Matake K, et al 2006 | Meeuwis C, et al 2010 | Moore W, et al 2010 | Morimoto T, et al 2008 | Piccolo D, et al 2002 | Sadik M, et al 2009 | Sahiner B, et al 2009 | Sawaki A, et al 1999 |
|  |  | Seidenari S, et al 1998 | Shen WC, et al 2007 | Shi X, et al 2010 | Stanganelli I,, et al 2005 | Szucs-Farkas Z,, et al 2010 | Taylor SA, et al 2008 | Van den Biggelaar FJHM,, et al 2010 | van Beek EJR,, et al 2008 | Vergnaghi D, et al 2001 | Wang Y, et al 2010 | Yu YH,, et al 2008 | Zhang J, et al 2007 |  |  |  |  |  |  |
| Zhao et al,  2019, | 5 | Jeong et al 2018 | Gitto et al 2018 | Yoo et al 2018 | Gao et al 2017 | Choi et al 2016 |  |  |  |  |  |  |  |  |  |  |  |  |  |
| Azavedo et al, 2012, | 4 | Gilbert et al., 2008 | Gromet et al., 2008 | Georgian-Smith et al., 2007 | Khoo et al., 2005 |  |  |  |  |  |  |  |  |  |  |  |  |  |  |
| Cuocolo et al, 2020, | 12 | Abraham et al  (2019 ) | Antonelli et al (TZ)  Antonelli et al (PZ)  2019 | Bonekamp et al  2018 | Chaddad et al  2018 | Chen et al  2019 | Dikaios et al (PZ)  Dikaios et al (TZ)  2015 | Fehr et al  2015 | Le et al  2017 | Li et al  2018 | Sobecki et al  2018 | Toivonen et al  2019 | Zhong et al  2019 |  |  |  |  |  |  |
| Xing et al, 2021, | 15 | Artan 2010 | Bonekamp 2018 | Giannini 2015 | Giannini 2016 | Kwak 2015 | Litjens 2014 | Liu 2013 | Puech 2007 | Roethke 2016 | Thon 2017 | Vos 2012 | Wang 2017 | Yang 2017 | Zhao 2015 | Zhong 2018 |  |  |  |
| Tabatabaei et al, 2021, | 18 | Cui et al., 2018d | Chen et al., 2020 | Xiao et al., 2019 | Wu et al., 2018 | Vamvakas et al., 2019 | Lu et al., 2018 | Hashido et al., 2020 | Cho et al., 2018 | Lin et al., 2017 | Cho et al., 2017 | Takahashi et al., 2019 | Park et al., 2019 | Wang et al., 2019 | Tian et al., 2018 | Wu et al., 2018 | Gao et al., 2020 | Çinarer et al., 2020 | Bi et al., 2019 |
| Henriksen EL  2018 | 13 | Dean JC 2006 | Ko JM 2006 | Morton MJ 2006 | Fenton JJ 2007 | Fenton JJ 2011 | Gomez SS 2011 | Fenton JJ 2013 | Lehman CD 2015 | Gilbert FJ 2008 | Romero C 2011 | Bargallo X 2014 | Georgian-Smith D 2007 | Gromet M  2008 |  |  |  |  |  |
