# Supplementary material for: The use of artificial intelligence tools in cancer detection compared to the traditional diagnostic imaging methods: An overview of the systematic reviews
Source: PLoS One. 2023 Oct 5;18(10):e0292063. doi: 10.1371/journal.pone.0292063 (PMC10553229; doi:10.1371/journal.pone.0292063)
Supplement: Attachment — Submitted filename: Response to Reviewers.doc [file pone.0292063.s005.doc]

Brasilia, February 28^th^, 2023

*Azhar Imran, Ph.D*

*Academic Editor*

*PLOS ONE*

RE: Manuscript No. PONE-D-22-32314

Dear Dr. Azhar,

We would like to thank you for the opportunity to revise our manuscript entitled **“The use of artificial intelligence tools in cancer detection compared to the traditional diagnostic imaging methods: an overview of systematic reviews”.** We are also grateful for the valuable and insightful considerations provided by the reviewers. We are sure that the changes resulting from their comments improved the final quality of our manuscript. As the staff editors considered that our manuscript fits the scope of the Early Detection, Screening and Diagnosis of Cancer Call for Papers, we agreed with this statement and included it in the cover letter, as requested.

All suggestions were accepted, and further comments are given in detail. Text changes are highlighted in yellow in the manuscript. We listed the responses to all the reviewer’s comments, hoping that the revised manuscript will be considered for publication after the modifications.

**Journal Requirements:**

1. **Comment:** Please ensure that your manuscript meets PLOS ONE's style requirements, including those for file naming.

**Our response:** Modifications were made to the manuscript in accordance with the submission rules of PLOS ONE's style requirements.

2. **Comment:** Our staff editors have determined that your manuscript is likely within the scope of our Early Detection, Screening and Diagnosis of Cancer Call for Papers. This editorial initiative is headed by in-house PLOS editors. This Call for Papers aims to explore recent advances in the early detection of cancer and implications of these advances for patient survival. Additional information can be found on our announcement page: https://collections.plos.org/call-for-papers/early-detection-screening-and-diagnosis-of-cancer/

**Our response:** The authors would like their manuscript to be considered for a call for papers on early detection, screening, and diagnosis of cancer. This request appears in the cover letter as suggested by the staff editors

3. **Comment:** For clarity purposes please amend your title so that it better reflects the study type and findings with something like 'The use of artificial intelligence tools in cancer detection compared to the traditional diagnostic imaging methods: A Systematic Review'

**Our response:** We agree with the reviewers’ concern. On the other hand, by definition, an Overviews of Reviews (Overviews) use explicit and systematic methods to search for and identify multiple systematic reviews on related research questions in the same topic area for the purpose of extracting and analysing their results across important outcomes^1,2^. Overviews are similar to reviews of interventions, but the unit of searching, inclusion and data analysis is the systematic review rather than the primary study^1^. Thus, as the paper consists of an overview of systematic reviews, we decided to change the title, following the reviewer’s suggestion and in order to better clarifiy that this is an overview of systematic reviews.

1. Pollock M, Fernandes RM, Becker LA, Pieper D, Hartling L. Chapter V: Overviews of Reviews. In: Higgins JPT, Thomas J, Chandler J, Cumpston M, Li T, Page MJ, Welch VA (editors). *Cochrane Handbook for Systematic Reviews of Interventions* version 6.3 (updated February 2022). Cochrane, 2022. Available from [www.training.cochrane.org/handbook](http://www.training.cochrane.org/handbook).
2. Thomson D, Russell K, Becker L, Klassen T, Hartling L. The evolution of a new publication type: Steps and challenges of producing overviews of reviews. Research Synthesis Methods. 2010;1(3-4): 198-211.
4. **Comment:** Thank you for stating the following financial disclosure:
   "The funders had no role in study design, data collection and analysis, decision to publish, or preparation of the manuscript." … Please include your amended statements within your cover letter; we will change the online submission form on your behalf.

**Our response:** Modifications regarding research funding and financial support from the institution are described in the cover letter as requested by the reviewers.

5. **Comment:** We note that you have stated that you will provide repository information for your data at acceptance. Should your manuscript be accepted for publication, we will hold it until you provide the relevant accession numbers or DOIs necessary to access your data. If you wish to make changes to your Data Availability statement, please describe these changes in your cover letter and we will update your Data Availability statement to reflect the information you provide.

**Our response:** Modifications regarding the provision of repository information are described in the cover letter as requested by reviewers.

**Reviewer #1:**

1. **Comment:**  How is this review paper different from other recently published articles (2021 and 2022)?

**Our response:** The authors' intention was to carry out an overview that gathered the knowledge already published by systematic reviews in the literature regarding the use of artificial intelligence in the detection of cancer and its comparison with traditional imaging diagnostic methods in a single publication, aggregating the main evidence from articles of greater scientific robustness. A justification for carrying out the overview was added in the Introduction section on page 6 in lines 158-160.

2. **Comment:** What do you mean by the traditional diagnostic imaging methods? Where did you provide the comparison?

**Our respons**e: It is the method that the radiologist/radiology resident uses, either by single reading or by double reading, to perform the evaluation of radiological images for cancer detection. The comparison can be found in the Session Discussion, page 24-25, lines 480-514.

3. **Comment:** There are lots of missed studies in cancer detection/prediction by ML/DL methods such as:**...**Could the authors explain why these studies are not reviewed?

**Our response:**  The overview carried out had as one of the inclusion criteria the use only of systematic review studies that addressed the relationship between the use of artificial intelligence tools in the detection of cancer and its comparison with traditional imaging diagnostic methods. This choice is due to the fact that the systematic review is a type of paper that is focused on a well-defined question, which aims to identify, select, evaluate and synthesize the relevant available evidence, with a critical analysis of the literature and a minimum of bias. The studies pointed out by Reviewer #1, despite addressing the subject of this overview, they are not systematic reviews to be selected according to the eligibility criteria listed in this overview. In addition, some studies used histological slides in the detection of cancer and the focus of the da overview was on the use of medical radiological images. Thus, they are not part of the selected studies.

4. **Comment:** Please discuss the most important existing cancer databases.

**Our response:** The Discuss of the most important existing cancer databases has been added to the Discussion Session on page 30-31, lines 634-657, as suggested by Reviewer #1

5. **Comment:** As this paper aims to highlight the impact of Machine Learning (ML) and Deep Learning (DL) methods in cancer detection, it is highly recommended that the authors provide more results and discussions on the proposed ML/DL methods (theoretical) and not just providing their names in general.

**Our response:**  Discussion of the impact of Machine Learning (ML) and Deep Learning (DL) methods in cancer detection is described in the Discussion section on page 27-28, lines 564-600 as suggested by Reviewer #1

6. **Comment:** Discussion: Which Machine Learning (ML) and Deep Learning (DL) methods are best in detecting cancers?

**Our response:**  The authors agree with reviewer #1 and the discussion of Which Machine Learning (ML) and Deep Learning (DL) methods are best in detecting cancers is in the Discussion section, on page 25-26, lines 520-548. And in the Conclusion section page 38, lines 864.

7. **Comment:** Please list and discuss the major limitations in the domain and literature.

**Our response:** Authors agree with reviewer #1 and limitations related to selected systematic reviews and limitations related to overview are described on pages 35-36 lines 772-795.

8. **Comment:** The authors are highly encouraged to provide a new section including main research gaps and future ML/DL research directions.

**Our response:**  The authors agree with Reviewer#1 and a new section including main research gaps and future ML/DL research directions added in Section Discussion on page 36-38 on lines 797-857.

9. **Comment:** The authors could discuss several important topics in the application of ML/DL methods in cancer detection. This means that discussions on uncertainty quantification and explainability (Explainable Artificial Intelligence (XAI)) of such methods should be considered. For more information the authors can use the following fully related studies:...

**Our response:**  The authors created two new subsections in the discussion section to discuss the two topics suggested by Reviewer #1. The subject of Explainable Artificial Intelligence (XAI) is in the subsection "The role of explainable artificial intelligence in DL and ML models" on page 31-33, lines 659-720. And the subject of uncertainty quantification is in the subsection "The use of uncertainty quantification approaches in medical imaging" on page 33-35, lines 722-770.

**Reviewer #2:**

1. **Comment:** In this review, the three terms, AI, radiomics, and CAD were used interchangeably somehow. I would suggest the author to explain the definitions and relationships of these three terms in the Introduction section, and avoid to mix these concepts.

**Our response**: The authors agree with reviewer #2 and a further explanation of the definitions and relationships of these three terms has been placed in the Introduction section on page 5-6 at lines 131-157.

2. **Comment:** In the Conclusions of the Abstract section, it reads: " When compared to standard diagnostic imaging, AI applications for cancer detection may be more accurate in adult patients". I understand this point was the aim for this overview. However, in the discussion and conclusion section of this review, I could not find similar and supporting statements for this point. Please add more discussions on this.

**Our response:** After reading the discussion session, the authors agreed with reviewer #2 and modified the conclusion of the abstract in order to make it more consistent with the final conclusions of the overview. Changes are on page 2-3, lines 49-55.

3. **Comment:** As the authors stated in the manuscript, the meta-analysis of the data was unfeasible because of the high methodological heterogeneity of these secondary research, I would suggest the authors to consider to summarize the primary studies on this topic for a meta-analysis.

**Our response:** Although the Cochrane Manual for Systematic Reviews of Interventions considers the possibility of meta-analysis for overviews, the authors wishing to re-analyse outcome data may only be able to do so if the clinical parameters and statistics aspects of the included systematic reviews are sufficiently reported^1^.

Due to the great heterogeneity of studies, data and types of cancer. In addition, three systematic reviews presented all the data, three systematic reviews presented partial data and three systematic reviews did not present enough data, even though the authors were requested by email and social networks, with no return response. Thus, the authors came to the conclusion that it would not be possible to carry out a meta-analysis of the primary studies of the selected systematic reviews. However, if the reviewers still deem it important and necessary to better qualify the knowledge presented in the overview, the authors ask for an extension of the deadline to carry out the meta-analysis of systematic reviews that present complete data.

1. Pollock M, Fernandes RM, Becker LA, Pieper D, Hartling L. Chapter V: Overviews of Reviews. In: Higgins JPT, Thomas J, Chandler J, Cumpston M, Li T, Page MJ, Welch VA (editors). *Cochrane Handbook for Systematic Reviews of Interventions* version 6.3 (updated February 2022). Cochrane, 2022. Available from [www.training.cochrane.org/handbook](http://www.training.cochrane.org/handbook).

We remain at your disposal for any further clarification that you may require regarding our manuscript. We forward to your reply. Thank you for your consideration.

Sincerely,

**Helbert Eustáquio Cardoso da Silva, in behalf of the all authors**

University of Brasilia, Health Sciences Faculty, Brasília, DF, Brazil.

e-mail: [helbertcardososilva@gmail.com](mailto:helbertcardososilva@gmail.com)
