# Supplementary material for: The use of artificial intelligence tools in cancer detection compared to the traditional diagnostic imaging methods: An overview of the systematic reviews
Source: PLoS One. 2023 Oct 5;18(10):e0292063. doi: 10.1371/journal.pone.0292063 (PMC10553229; doi:10.1371/journal.pone.0292063)
Supplement: Attachment — Submitted filename: Response to Reviewers.doc [file pone.0292063.s006.doc]

Brasilia, June 22^th^, 2023

*Azhar Imran, Ph.D*

*Academic Editor*

*PLOS ONE*

RE: Manuscript No. PONE-D-22-32314

Dear Dr. Azhar,

We would like to thank you for the opportunity to revise our manuscript entitled **“The use of artificial intelligence tools in cancer detection compared to the traditional diagnostic imaging methods: an overview of systematic reviews”.** We are also grateful for the valuable and insightful considerations provided by the reviewers. We are sure that the changes resulting from their comments improved the final quality of our manuscript. As the staff editors considered that our manuscript fits the scope of the Early Detection, Screening, and Diagnosis of Cancer Call for Papers, we agreed with this statement and included it in the cover letter, as requested.

All suggestions were accepted, and further comments are given in detail. Text changes are highlighted in yellow in the manuscript. We listed the responses to all the reviewer’s comments, hoping that the revised manuscript will be considered for publication after the modifications.

**Corrections by authors:**

The authors, after another careful reading, made some changes to the text of the manuscript, which will be highlighted below, with the aim of improving the understanding of the text, without changing the structure already approved by the reviewers.

The Background and purpose in the Abstract section, lines 28 to 30, has been replaced by the following sentence: "In comparison to conventional medical imaging diagnostic modalities, the aim of this overview article is to analyze the accuracy of the application of artificial intelligence (AI) techniques in the identification and diagnosis of malignant tumors in adult patients."

The expression "are more likely" was replaced by "are prone" on line 113.

The expression "systematic review" has been replaced by "overview" on line 172.

The following sentence was added in the Materials and methods section, in lines 174 to 176: "and was developed according to the JBI Manual for Evidence Synthesis (https://synthesismanual.jbi.global) and the Cochrane Handbook for Systematic Reviews (www .training.cochrane.org/handbook)."

The sentence was rewritten in the section Materials and methods, in lines 177 to 179: "The definition of systematic reviews considered was that established by the Cochrane Collaboration. A study was considered a systematic review when reporting or including:"

The sentence was rewritten in the Materials and methods section, in lines 258 to 262: " It should be noted that critical appraisal/risk of bias tools classically indicated for systematic reviews, such as AMSTAR 2 and ROBIS, were designed for systematic reviews of intervention, while the articles included were systematic reviews of diagnostic accuracy. We opted for performing the methodological assessment, not the risk of bias in the selected studies."

In the Results section, the initials RS were replaced by the initials SR due to a spelling error in lines 284, 285 and 286.

The expression "quality" was replaced by "certainty" on line 446.

In the Discussion section, the abbreviation ML was replaced by the expression "Machine learning" for a better understanding of the sentence, on line 570.

In the Discussion section, on line 850, the term "possible" was added in the sentence "possible presence of publication bias due to lack of studies with unfavorable data" for a better understanding of the sentence.

**Reviewer #2:**

1. **Comment:** As suggested by my previous comment 3, I think the authors should take more time to carry out the meta-analysis of systematic reviews that present complete data on this important topic.

**Our response**: We appreciate the suggestion and have considered it carefully. However, neither the Cochrane Handbook for Systematic Reviews^1^ nor the JBI Manual for Evidence Synthesis^2^ (used as the main guidance for conducting this overview) foreseen a quantitative synthesis for Overviews/Umbrella Reviews. Both suggest only a critical evaluation of the meta-analyses presented by the systematic reviews included in the overview.

In addition, the studied meta-analyses presented some limitations. First, all showed considerable heterogeneity with regard to sensitivity and specificity across studies. It is likely that this heterogeneity is related to methodological aspects of the different studies, and to the basic differences between the included patients. Second, the sample size of the included studies was relatively small (Dorrius 2011 and Zhao 2019, Eadie 2012). The authors considered the occurrence of selection (Dorrius 2011 and Zhao 2019, Eadie 2012), measurement (Zhao 2019) and publication (Zhao 2019, Eadie 2012) bias in performing the meta-analyses.

As a solution discussed by the authors, we decided to carry out a critical analysis of the only three studies that presented complete data (Dorrius et al., 2011, Zhao et al., 2019, Eadie et al., 2012), included in the Discussion section, lines 664 to 726, as a way of giving the closest answer to the reviewer's #2 request and consistent with what the literature recommends regarding performing an overview.

^1^Higgins JPT, Thomas J, Chandler J, Cumpston M, Li T, Page MJ, Welch VA (editors). Cochrane Handbook for Systematic Reviews of Interventions version 6.3 (updated February 2022). Cochrane, 2022. Available from www.training.cochrane.org/handbook.

^2^Aromataris E, Munn Z (Editors). JBI Manual for Evidence Synthesis. JBI, 2020. Available from [https://synthesismanual.jbi.global](https://synthesismanual.jbi.global/). [https://doi.org/10.46658/JBIMES-20-01](https://jbi-global-wiki.refined.site/space/MANUAL)

We remain at your disposal for any further clarification that you may require regarding our manuscript. We look forward to your reply. Thank you for your consideration.

Sincerely,

**Helbert Eustáquio Cardoso da Silva, on behalf of the authors**

University of Brasilia, Health Sciences Faculty, Brasília, DF, Brazil.

e-mail: [helbertcardososilva@gmail.com](mailto:helbertcardososilva@gmail.com)
